# A Critical Assessment of Geographic Clusters of Breast and Lung Cancer Incidences among Residents Living near the Tittabawassee and Saginaw Rivers, Michigan, USA

**DOI:** 10.1155/2009/316249

**Published:** 2009-11-25

**Authors:** Olga A. Guajardo, Tonny J. Oyana

**Affiliations:** Advanced Geospatial Analysis Lab, Department of Geography and Environmental Resources, Southern Illinois University, 1000 Faner Drive, MC 4514, Carbondale, IL 62901-4514, USA

## Abstract

*Objectives*. To assess previously determined geographic clusters of breast and lung cancer incidences among residents living near the Tittabawassee and Saginaw Rivers, Michigan, using a new set of environmental factors. *Materials and Methods*. Breast and lung cancer data were acquired from the Michigan Department of Community Health, along with point source pollution data from the U.S. Environmental Protection Agency. The datasets were used to determine whether there is a spatial association between disease risk and environmental contamination. GIS and spatial techniques were combined with statistical analysis to investigate local risk of breast and lung cancer. *Results and Conclusion*. The study suggests that neighborhoods in close proximity to the river were associated with a high risk of breast cancer, while increased risk of lung cancer was detected among neighborhoods in close proximity to point source pollution and major highways. Statistically significant (*P* ≤ .001) clusters of cancer incidences were observed among residents living near the rivers. These findings are useful to researchers and governmental agencies for risk assessment, regulation, and control of environmental contamination in the floodplains.

## 1. Introduction

Lung cancer is the leading cause of cancer-related death in Michigan and in the United States (U.S.), while breast cancer is the most frequently diagnosed cancer among Michigan women and the leading cause of death in the U.S. women between their late thirties and early fifties [1, 2]. 

Studies on breast cancer suggest that breast cancer involves a complex interaction of internally (endogenous) and externally (exogenous) introduced factors [3]. The most important factors include aging, genetics, race, not having children or having them later in life, and lack of exercise [4–7]. In addition, recent epidemiologic and animal studies provide emerging evidence of association between breast cancer risk and environmental contamination [1, 4–11]. Specifically, laboratory studies revealed 216 potential mammary carcinogens identified in animals and 250 estrogen mimics [7]. To date the most suspecting pollutants are persistent organochlorine compounds, organic solvents, polycyclic aromatic hydrocarbons (PAHs), dioxins and furans (including polychlorinated biphenyls (PCBs)), disinfection byproducts, and organochlorine pesticides [4, 6, 8–11]. Still, the evidence of association between exposure to these pollutants and breast cancer is rather inconsistent and requires further investigation.

Furthermore, some studies have noted differences in breast cancer incidences based on ethnicity, education level, and years of residence at the same location [1, 9, 11, 12]. In particular, the risk for breast cancer was suggested to be higher among the white population in comparison to African American, Hispanics, and Asian Americans, with education level beyond the high school, and with longer years of residence at the same location, while lowest rates were found among women living in Asian countries and in American Indian and Alaska Native women [1, 9, 11–14].

The spatial variations of breast cancer occurrence suggest a potential hypothesis partially due to local and regional environmental risk factors [4, 9, 11, 15]. Specifically, recent studies conducted in the U.S. revealed increased breast cancer incidences and elevated levels in the northeastern and western parts of the country that are partly due to higher developed industry and more intense traffic [1, 11]. Owing to complex spatiotemporal variations, additional risk factors for breast cancer still require further investigation. Both basic and empirical studies should be done to investigate whether there is a strong association between environmental exposure and breast cancer.

Lung cancer on the other hand involves several factors some of them include cigarette smoking, personal and family health history, and environmental pollution. Cigarette smoking, which accounts for 87 percent of all lung cancers, is recognized as the leading risk factor for lung cancer [16]. Other factors that increase the likelihood of having lung cancer include radiation treatment to the lungs; personal and family history; genetics; diet and vitamins; air pollution, such as 1,2-dichloroethane, arsenic (inorganic compounds), asbestos, benzene, beryllium, cadmium, chloromethyl ethers, chromium compounds, coal products, dichloromethane, dioxins, fine particulates, mustard gas, naphthalene, nickel compounds, PAHs, radionuclides, radon, trichloroethylene (TCE), uranium, vinyl chloride; and diesel exhaust [2, 16–18]. The primary sources of many of these organic and inorganic compounds, oxidants, and acids include combustion of fossil fuels for power generation or transportation [19].

Due to emerging evidence of effects of air pollution on lung cancer there is an urgent need to conduct additional studies to establish a casual relationship between environmental pollution and lung cancer. Recent studies on lung cancer and environmental pollution suggest that urban/rural differences in lung cancer incidence is due to presence of a variety of known and potential human carcinogens in polluted air of urbanized areas [17, 19–21].

Studies on socioeconomic risk factors suggest that race and education may also contribute to a higher risk of lung cancer. In several studies, for example, a higher risk of lung cancer was found among African Americans with lower education level and Whites as compared to recent immigrants from India and Africa, which may be attributed to smoking [20, 22–25].

Although air pollution appears to be a minor contributor to lung cancer compared to smoking, it involuntarily affects a significant population and therefore requires greater attention. Growing evidence of an association between a lung cancer and environmental exposure reflects the importance of the need to assess its relative contribution [18, 19, 23–26]. Findings from new studies may help generate stronger strategies towards reduction of lung cancer, especially among the nonsmoking population. 

The availability of large surveillance datasets and spatial techniques makes it easy to analyze breast and lung cancer data relative to environmental factors. In particular, Geographic Information Systems (GISs) are an emerging tool in environmental epidemiology studies, especially for cancer studies [27, 28]. GIS techniques can generate effective exposure and disease models for assessment of spatial patterns and serve as effective tools for exploration and communication of cancer data [29].

Since few studies have addressed the analysis of risk factors of breast and lung cancer incidences in the floodplains of the Tittabawassee and Saginaw Rivers, the overarching goal of this study is to determine whether there is a connection between environmental exposure and cancer disease by critically examining a priori clusters of cancer disease in Michigan State. The study hypothesizes that there is a potential link between increased risks of cancer disease and sources of pollution suspected to be environmentally contaminated. Three research questions were formulated to help with investigating the hypothesis. (1) Are there any environmental contaminants in the floodplains of the Tittabawassee and Saginaw Rivers that have a potential to contribute to breast and lung cancer within the at-risk population? (2) Is there a relationship between identified environmental contaminants and breast and lung cancer incidences in the study area? (3) Is there a relationship between spatial proximity to point source pollution and cancer incidence? To answer these questions we use geographically based exposure assessment strategies. The study employed GIS and spatial techniques combined with statistical analysis to examine occurrence of cancer incidences in relation to proximity from point source pollution, major highways, and the floodplains of the Tittabawassee and Saginaw Rivers.

## 2. Materials and Methods

### 2.1. Study Design

This retrospective study covers a period extending 14 years between 1989 and 2002 to investigate the occurrence of breast and lung cancer in relation to environmental contamination. The study suspects a major health risk due to the exposure of pollution sources. Therefore, the study design was focused on critically assessing previously determined geographic clusters of breast and lung cancer incidences among residents living near the Tittabawassee and Saginaw Rivers, Michigan, using a new set of environmental factors [30, 31]. Breast and lung cancer incidence rates were derived at the ZIP code level and adjusted by age using the 2000 U.S. Census data. In subsequent analyses, a variety of spatial models were constructed to explain disease risk and potential trends.

### 2.2. Study Area

**Figure 1:**
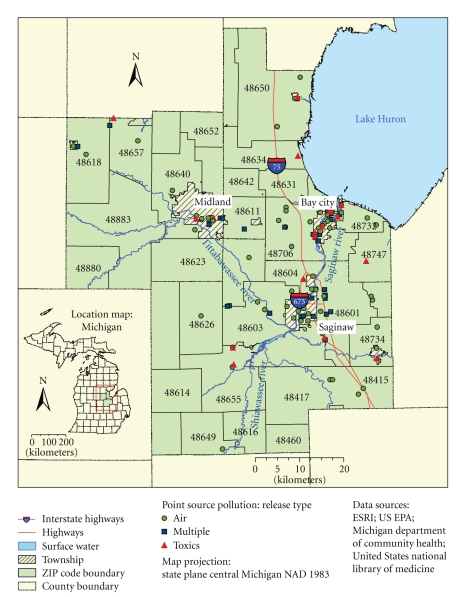
Map showing the location of the study area and point source pollution, grouped by emission release type; major roads; and surface water, including the Tittabawassee and Saginaw Rivers.

The study area consists of 38 ZIP codes, located within the Bay, Midland, and Saginaw counties, central Michigan (Figure 1). According to the U.S. Census Bureau, total population in the study area in the year 2000 was 417 423 [32]. The study area mainly encompasses three major communities: Midland (population about 42 000), Saginaw (population about 62 000), and Bay City (population about 37 000) with total population approximately 156 000 [32].

Potential risk factors of cancer in this area comprise a variety of components of physical and built environments including industry (i.e., chemical production, wood treatment, petroleum production, furniture refinishing, cement production, solid waste incineration, waste water treatment, casting production, and power generation), major highways, and hydrological infrastructure (i.e., the Tittabawassee and Saginaw River, and Saginaw Bay of Lake Huron) [33]. About half of the land use within major communities is used for industrial production, while the land use outside of these communities is primarily rural residential and is characterized by small-scale agricultural production. A variety of public recreational facilities, including parks, boat launches, and public access sites are located within the floodplain of the Tittabawassee River. The composition of the study population is as follows: Whites (83.5%), followed by Blacks or African Americans (10.4%), Hispanics (4.8%), Asian (0.8%), and Native Americans (0.5%). More than 80% of the population consists of high school graduates, and 98% of the residents are native born.

### 2.3. Data Categories and Processing

Four data categories were used to analyze the spatial risk of disease: disease data (annual breast and lung cancer incidences, 1989–2002), sources of environmental pollution (industrial facilities and major highways), the 2000 U.S. Census data, and baseline data (major cities, major roads, surface water, and ZIP code boundaries).

Data pertaining to cancer were acquired from the Michigan Department of Community Health (MDCH), Vital Records and Health Statistics Unit, Development Section [34]. The point source pollution data, including facility location and release type, were obtained from the U.S. Environmental Protection Agency (U.S. EPA) [33]. Additional information on released chemicals and activity status over the study period for each industrial facility was gathered from the U.S. National Library of Medicine database [35]. The census data were obtained from the U.S. Census Bureau [32]. The baseline data were compiled from the Michigan Department of Environmental Quality (MDEQ), the Michigan Center for Geographic Information (MCGI), and partially from the Environmental Systems Research Institute (ESRI). 

#### 2.3.1. Disease and Census Data

The disease data represent a list of annually diagnosed cancer cases (incidences) from the MDCH cancer registry database, which complies with quality standards of the National Cancer Institute's Surveillance, Epidemiology, and Results (SEER) Program. Each data record contains invasive cancer case, patient's ZIP code of residence at diagnosis, patient's gender, type of diagnosed cancer, and patient's age group. Since protecting the patient's privacy is required by law, the MDCH only provides ZIP code referenced cancer data where there are more than 5 000 people. Although this spatial unit is coarse, it was the best data available to support this level of analysis. Additionally, cancer data can easily be linked to census data to provide additional insights regarding potential trends and spatial patterns.

In total, we had cancer data for twenty two ZIP codes containing numerous breast cancer cases (*n* = 3 768) and lung cancer cases (*n* = 4 014). Additionally, the original classification of cancer was recorded for six age groups: less than 15; 15 to 29; 30 to 44; 45 to 64; 65 to 74; and over 75 years of age. 

The 2000 U.S. Census data contains general characteristics, such as total population, gender, age, and race; as well as socioeconomic characteristics, including median household income, education level, and nativity. The census age groups were synchronized with the age groups of the cancer data. The census data were used as a denominator for calculating the incidence rates.

#### 2.3.2. Sources of Environmental Pollution

Point source pollution data represent locations of industrial facilities in the study area, classified according to the release type as air, toxics, and multiple, where multiple releases refer to combination of different release types (e.g., air and toxics) [33]. A total of 128 industrial facilities have been identified in the study area, including 79 facilities of air, 19 facilities of toxic, and 30 facilities of multiple release type. 

The physical addresses of industrial facilities, representing point source pollution, were geocoded (e.g., assigned latitude and longitude information) and mapped using ESRI's ArcGIS 9.2 (ESRI Inc, Redlands, California). Address matching was based on ESRI's USA street map data using U.S. Alphanumeric Ranges with Zone address locator style. To achieve a high success rate in assigning geographic coordinates and for quality control purposes, we performed a combination of automatic and interactive matching and crosschecking of addresses with a full user control over the entire process. As a result, 92% of addresses were matched with a score of 80 and higher, and 3% of addresses were matched with candidates tied. Figure 1 shows geocoded geographic locations of industrial facilities grouped by emission release type.

Based on the results of exploratory analysis, several point pollution sources were selected to analyze the decay distance-exposure relationship between location of selected sources and cancer incidence rates. Specifically, selection of pollution sources (industrial facilities) was based on numerous criteria, including history of carcinogenic releases during the study period; and location of facility in the ZIP code characterized by low or high cancer incidence, and within a spatial cluster detected using spatial techniques. As a result of these criteria and exploratory analysis, 16 out of 128 industrial facilities were selected for thorough investigation. Also six of these facilities have been identified in recent studies as potential sources responsible for increased risk of breast cancer (Table 1), and ten facilities as potential sources responsible for increased risk of lung cancer (Table 2). The chemical composition for potential sources that may be responsible for the increased risk of breast cancer includes benzene, chlorinated solvents, chloroprene, dioxins, organochlorine pesticides, and PAHs; for lung cancer there are 1, 2-dichloroethane, arsenic (inorganic compounds), benzene, chromium compounds, dichloromethane, dioxins, naphthalene, nickel compounds, PAHs, and TCE.

### 2.4. Data Analysis

Data analysis consisted of three major aspects: preliminary data analysis (e.g., descriptive statistics) and GIS mapping (e.g., disease maps), statistical techniques (e.g., odds ratio statistics and discriminant analysis), and spatial analysis (e.g., smoothing techniques and spatial cluster statistics: Local Moran's test, the Turnbull's method, the Lawson and Waller score test, and Bithell's linear risk score test). 

The analysis was conducted using numerous software applications, including ArcGIS 9.2, ArcView 3.3, GeoDa 0.95i (University of Illinois, Urbana-Champaign, Illinois); ClusterSeer 2.0 and TerraSeer's STIS 1.6 (TerraSeer Inc., Ann Arbor, Michigan); Microsoft Excel (Microsoft Inc., Redmond, Washington); and SPSS 17.0 (SPSS Inc., Chicago, Illinois). 

#### 2.4.1. Preliminary Data Analysis and GIS Mapping

Preliminary analysis was done to explore, describe, and summarize the data, detect outliers and anomalies, or to detect relationships and generate hypotheses regarding the data. Basic statistics was generated to summarize the data using the mean, standard deviation, coefficient of variation, and 95% confidence level. The data were mapped to show spatial patterns and distributions of industrial facilities with regards to the cancer data.

#### 2.4.2. Statistical Techniques

**Figure 2:**
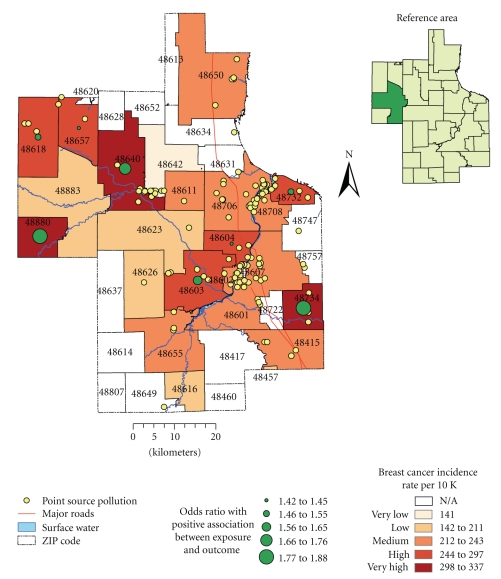
Breast cancer incidence rates per 10 000 females and odds ratio illustrating positive association between breast cancer incidences among female residents living near suspected sources of environmental contamination covering the period 1989–2002.

**Figure 3:**
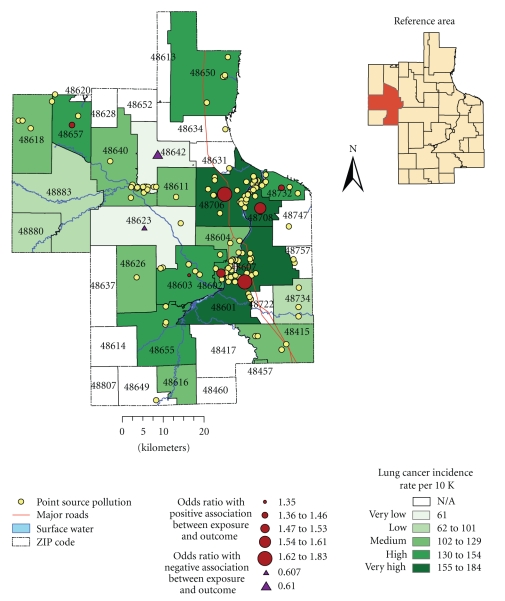
Lung cancer incidence rates per 10,000 people and odds ratio illustrating positive association between lung cancer incidences among residents living near suspected sources of environmental contamination covering the period 1989–2002.

The odds ratio statistics at a significance level of *P* ≤ .05 were employed to test whether there is a significant association between exposure to environmental pollution and outcome between exposed and unexposed groups: the cases and controls, correspondingly. The population in ZIP code 48883 (reference area in Figures 2 and 3) was assumed to be unexposed to environmental pollution and served as the control group for odds ratio analysis. This reference area was selected because it is located farther away from the sources of environmental pollution, such as industrial facilities, major highways, and the floodplains of the Tittabawassee and Saginaw Rivers; it is also characterized by low incidences of both breast and lung cancer. The odds for having cancer were calculated using a 2 × 2 contingency table with 95% confidence interval (CI). The CI was calculated using a formula suggested by Woolf [36]. 

Discriminant function analysis, using a stepwise method, was performed to determine the relative importance of socioeconomic risk factors in relation to the spatial variability of cancer. Socio-economic variables selected for the analysis include median household income (dollars per year), race (percent of White, Black, Hispanic, Native American and Asian), nativity (percent of native born), education level (percent of high school graduates), and residency (percent of population residing at the same address in 1995). To complete this analysis, ZIP codes with synthesized spatial clusters of breast and lung cancer were classified as areas of high disease risk and ZIP codes without clusters of cancer as areas of low or no disease risk.

#### 2.4.3. Spatial Analysis

The ordinary kriging of cancer incidence data was performed to account for spatial variations within the data, to reduce impact of small population size or uncertainty, and because the rates were strongly positively skewed [37–39]. The kriged incidence data (e.g., derived from the model) represent precise prediction (estimate) of unkriged (e.g., measured data, not derived form the model) data pairs within the sampling unit. The popularity of the Empirical Bayesian Smoothing technique also motivated us to try this method; however in using both of these smoothing techniques to account for the small population problem we did not observe any statistically significant differences in incidence rates. Both methods worked well. 

Four spatial techniques were used to identify clusters of increased cancer incidence rates, where a cluster represents any area within the study region of significant elevated risk of disease occurrence [40]. Employed methods include two general methods: global (e.g., Local Moran's test) and local (e.g., Turnbull's method); and two focused methods: the Bithell's linear risk score test and the Lawson and Waller score test. Local Moran's test, with randomization of the data 9 999 times due to small sample size, was used to confirm the presence of spatial patterns of breast and lung cancer and to determine existence of cancer clusters. Turnbull's method was used to detect local spatial clusters at group level (e.g., determine areas of significant increased risk of cancer). The Lawson and Waller score test was used to evaluate if industrial facilities, identified as potential sources of increased cancer risk, were associated with increased risk of breast and lung cancer. The Lawson and Waller score test examines the decay distance-exposure relationship in group level data. Bithell's linear risk score test investigates whether there is a cluster of cases around the identified location.

## 3. Results and Discussion

### 3.1. Results

#### 3.1.1. Overall Demographics of Cancer Incidences

In the MDCH database there were 3 769 female breast cancer cases and 4 014 lung cancer cases registered over 14 years of the study period. The majority of both breast and lung cancer incidences (e.g., approximately 88% of breast cancer and 97% of lung cancer cases) were adults over 45 years of age. Less than one percent of cancer patients were younger than the age of 30 (e.g., 0.53% of breast cancer and 0.27% of lung cancer cases). There were no female breast cancer patients younger than the age of 15, and only one breast cancer patient was of unknown age, whose record was excluded from the analysis.

**Breast Cancer**

The highest average rate of breast cancer was recorded in 2000 during the study period, while the year 1998 was characterized by the lowest average rate of breast cancer. There was a notable difference between the mean incidence rate and standard deviation suggesting wide dispersion in the observations. The CI was relatively narrow, confirming overall accuracy of the breast cancer data. The temporal trend portrayed a stable progression of average annual rates; yet it was characterized by presence of a distinguishable peak of high incidence rate in 2000. Using the total number of cases as the denominator, the highest incidences were from ZIP codes 48603 (13.7%), 48601 (11.6%), 48640 (11.2%), and 48706 (10.8%), mainly located within Midland and Saginaw communities (Figure 2). The female population over 45 years of age, and particularly females between 45 and 64 years of age, had the highest rates. After adjusting for age, the females between 65 and 75 had the highest rates.

**Lung Cancer**

The highest average rate of lung cancer was recorded in 2000 during the study period, while the year 1990 had the lowest average rate of lung cancer. There was a notable difference between the mean incidence rate and standard deviation suggesting wide dispersion in the observations. The CI was relatively narrow, confirming overall accuracy of lung cancer data. The temporal trend was characterized by a slight increase. Moreover, there was a considerable increase incidence rates between 1990 and 1994 with an obvious peak in 2000. Using the total number of cases as the denominator, the highest incidences were from ZIP codes 48601 (15.8%), 48706 (14.3%), and 48603 (11%), located within Saginaw and Bay City communities (Figure 3). Population over 45 years of age and particularly in the age group between 45 and 74 years old had the highest rates, with slightly declining trend among patients over 75 years. After adjusting for age, the population between 65 and 75 years old had the highest incidence rates.

#### 3.1.2. Analysis of Cancer Risk and Environmental Exposure

**Spatial Patterns of Breast Cancer Incidences**

The incidence rates were derived for the female population who were 15 years and older as the denominator; the overall rates ranged from 141 to 337 for every 10 000 females. The rates were highest among female residents living in ZIP codes 48734, 48880, 48640; and higher in 48603, 48618, 48732, 48706, and 48604 (Figure 2). These ZIP codes are the major population centers/neighborhoods in the study region (e.g., Midland, Saginaw and Bay City townships) and are in close proximity to the Tittabawassee and Saginaw Rivers. Even after adjustments, the general trend of spatial distribution of cancer incidence was persistent suggesting the presence of one of the highest rates in Midland Township as well as near the Saginaw River.

**Spatial Patterns of Lung Cancer Incidences**

The incidence rates were calculated using population who are 15 years and older as the denominator; the overall rates ranged from 61 to 184 per every 10 000 people. The incidence rates were highest among neighborhoods located in ZIP codes 48601, 48706, 48708; and higher in 48602, 48732, 48657, 48655, 48603, and 48650 (Figure 3). These are mainly located in southeastern portion of the study region. However, after applying smoothing techniques, some of the spatial patterns in the southeastern portion remained persistent. This area is also known to have a large presence of major industrial facilities and highways.

**Spatial Patterns of Breast and Lung Cancer Incidence**

A closer examination of both the breast and lung cancer incidence rates suggests the presence of consistent nonhomogenous spatial patterns in the study region. Specifically, the female adult populations with increased risk of breast cancer reside in close proximity to the floodplains of the Tittabawassee and Saginaw Rives, while those with increased risk of lung cancer seem to reside near industrial facilities and major highways.

#### 3.1.3. Assessment Using Odds Ratio Analysis

**Breast Cancer and Lung Cancer**

Table 3 shows odds of having breast and lung cancer at the ZIP code level. A positive association between possible exposure to environmental contamination and breast cancer (at significance level *P* ≤ .05) was found in 40% of ZIP codes (e.g., in female populations residing in ZIP codes 48734, 48880, 48640, 48603, 48618, 48732, 48657, and 48604).

A map of these odds ratio shows a significant association between exposure and outcome and is in agreement with spatial patterns of breast cancer incidence rates that were observed earlier confirming increased breast cancer risk around the floodplains of major rivers (Figure 2).

For lung cancer, a positive association between possible exposure to environmental pollution and the lung cancer was observed in 32% of ZIP codes (e.g., in populations residing in ZIP codes 48601, 48706, 48708, 48602, 48732, 48657, 48603, and 48650). However, populations residing in ZIP codes 48642 and 48623 had a negative association.

A map of these odd ratios shows a significant association between exposure and outcome thus confirming increased risk near industrial facilities and major highways, which are located in southeastern portion of the study region (Figure 3). A negative association was detected in the areas that are located farther away from major industry.

#### 3.1.4. Assessment Using Empirical Bayesian Smoothing and Local Moran's Test

When Empirical Bayesian Smoothing was applied, high incidence rates of breast cancer incidences were observed in ZIP codes 48640, 48603, and 48734. For lung cancer, the high incidence rates were observed in ZIP codes 48732, 48706, 48708, and 48601. 

None of the breast cancer incidence rates were statistically significant after a Local Moran's test was done, while only two ZIP codes 48732 and 48708 were statistically significant for lung cancer. These observations are pretty consistent with what we have observed with other techniques. Overall, these techniques provide preliminary evidence of high incidence rates in some geographic locations in the study region, which seem to be persistent.

#### 3.1.5. Assessment Using Turnbull's Method

The assessment of cancer rates using Turnbull's method revealed additional insights regarding the nonhomogenous nature of the spatial distribution of both breast and lung cancer. 

For breast cancer, we investigated clusters within a population of 7 105 females at 95% confidence level. The method detected two statistically significant clusters of increased breast cancer incidence rates among residents living in ZIP codes 48640 (first most likely cluster at *P*-value = .0006) and 48603 (second most likely cluster at *P*-value = .00164), located within Midland and Saginaw communities situated at the beginning of the Tittabawassee and Saginaw Rivers, respectively. 

While for lung cancer we investigated clusters within a population of 13 504 people at 99% CI. We detected two statistically significant clusters of increased lung cancer incidence rates in ZIP codes 48602 (first most likely cluster at *P*-value = .0001) and 48706 (second most likely cluster at *P*-value = .0002), located within Saginaw community (upstream of Saginaw River) and Bay City township (downstream of the Saginaw River, where it empties into Lake Huron), respectively.

#### 3.1.6. Assessment Using Bithell's Linear Risk Score and Lawson and Waller Score Tests

**Breast Cancer**

The Lawson and Waller score test was based on 9 999 Monte Carlo simulations with 99% CI and none of the sites were statistically significant. Even with Bithell's linear risk score test there were no statistically significant sites.

**Lung Cancer**

**Figure 4:**
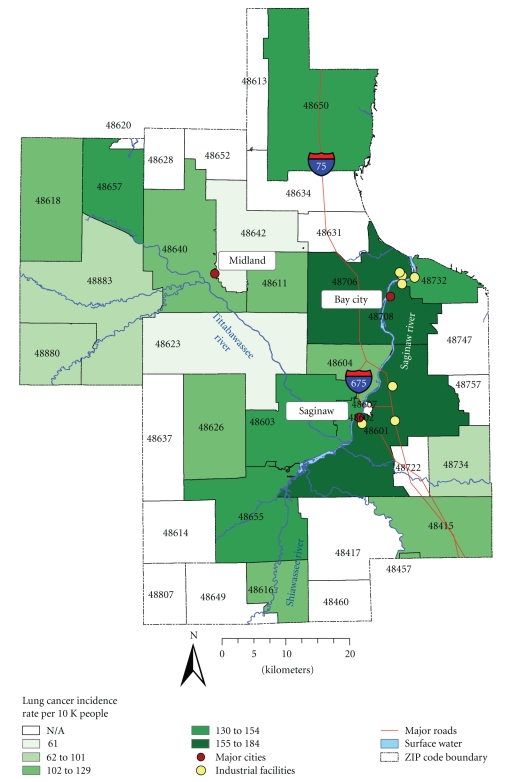
Locations of industrial facilities associated with increased risk of lung cancer identified by Lawson and Waller score test, around which clusters of lung cancer have been detected, as presented in Table 4.

Table 4 shows results of the Lawson and Waller score test for lung cancer, which was based on 9 999 Monte Carlo simulations with 99% CI. For this cancer, we detected statistically significant results for several industrial facilities suggesting a significant elevated risk of lung cancer around key sources of pollution. Specifically, we found significant clusters of lung cancer around seven industrial facilities, which are located in ZIP codes 48601, 48706, 48708, and 48732 (see Figure 4 and Table 4).

The industry types of these facilities include motor vehicle parts and accessories, automotive stampings, business services, petroleum bulk stations and terminals, wood preserving, and cement production. Reported carcinogenic compounds released from these facilities include arsenic, benzene, chromium, dichloromethane, naphthalene, nickel, TCE, and PAHs. Almost all of these facilities, except for Essroc Cement Corporation and Means Industries Inc. Saginaw Facility, were reported to be operationally active as far back as 2002. This test confirms that there is an increased risk of lung cancer near industrial facilities.

**Figure 5:**
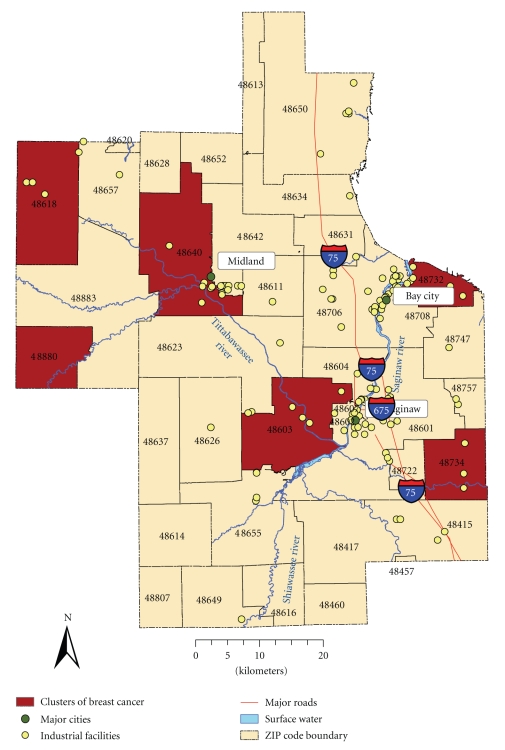
Synthesis of spatial clusters of breast cancer based on odds ratio analysis and Turnbull's method.

**Figure 6:**
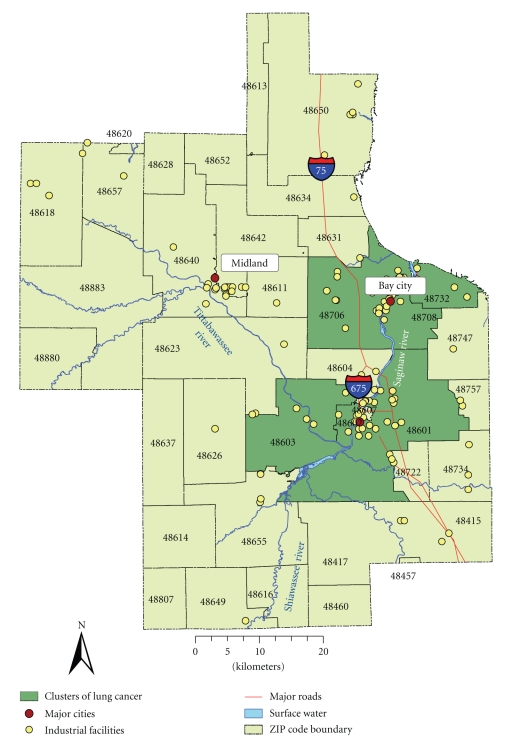
Synthesis of spatial clusters of lung cancer based on local Moran's test, odds ratio analysis, Turnbull's method, and Lawson and Waller score test.

In general, these focused tests revealed nonhomogeneous distribution of clusters of breast (Figure 5) and lung cancer (Figure 6) as was evident in earlier analyses. Although the findings for breast cancer were negative, the risk for breast cancer was still evident in the floodplains. The lack of a positive signal for breast cancer requires further investigation.

#### 3.1.7. Assessment of Risk Factors Using Discriminant Analysis

The areas identified with increased risk of breast and lung cancer were delineated to investigate the relative importance of socioeconomic characteristics. The discriminant analysis of risk areas of both breast and lung cancer successfully differentiated between high- and low-risk areas of cancer (*P*-value ≤.01). According to this analysis, the main risk socioeconomic factors for both breast and lung cancer in this study region was Hispanic race (for both breast and lung cancer) and residency at the same location (for breast cancer only).

### 3.2. Discussion

This study examined whether there is a spatial association between environmental pollution and incidences of breast and lung cancer among residents living in three counties of central Michigan. The major findings of this study are as follows:

1. the spatial distribution of breast and lung cancer incidence rates is nonhomogeneous;
2. a significant positive association between possible exposure to environmental pollution and risk of breast and lung cancer was found;
3. the spatial clusters of breast cancer were detected in locations that are in close proximity to the floodplains of major rivers, and spatial clusters of lung cancer were detected in locations that are in close proximity to point source pollution and major highways;
4. there are environmental contaminants with a potential to cause breast and lung cancer within at-risk population;
5. the socioeconomic factors, such as race and residency at the same location, are more likely to explain spatial variability of cancer incidences.

Preliminary analysis also confirmed results from previous studies, which suggests that aging is one of the risk factors [4–7, 21]. In this study, we found high breast and lung cancer incidences and incidence rates commonest among adults over 45 years old. Also, the analysis of temporal trends of cancer data identified the year 2000 with the highest incidence for both breast and lung cancer.

As was expected, there was no significant increase of average annual rates of breast cancer during the study period, which, as suggested by recent studies, is believed to be mostly due in part to better screening, awareness campaign, and improved treatment [15]. Lung cancer data, however, revealed a slight increase of annual average rates during the study period, especially in the early 1990s.

The analysis of these major findings suggests a spatial variation of the risk of breast and lung cancer within the study area. In general, the areas located in close proximity to major rivers were associated with elevated levels of breast cancer, and areas near to industry and major highways were associated with increased risk of lung cancer. Specifically, elevated rates of breast cancer incidence were found among populations living in ZIP codes 48640, 48603, 48734, 48880, 48618, and 48732. The ZIP codes 48640 and 48603 are located in close proximity to the rivers and to industrial facilities that may potentially be responsible for environmental contamination of the rivers. These results are consistent with findings from previous studies in central Michigan [30, 31]. Specifically, Dai and Oyana examined the variation of breast cancer using Kulldorff's spatial scan statistics, the improved genetic algorithm for spatial clustering, and Poisson regression model [31]. In their study, Dai and Oyana [31] considered soil dioxin contamination sites as the primary environmental risk factor for breast cancer. Based on the results of Poisson regression model, Dai and Oyana found a higher disease burden in ZIP codes 48640 and 48602 located in close proximity to soil dioxin contamination and based on results of Kulldorff's spatial scan statistics and improved genetic algorithm, Dai and Oyana identified spatial clusters of breast cancer located in the following ZIP codes: first 48640, 48603, and 48623 (located within major communities and in close proximity to soil dioxin contamination sites); second 48734; and third 48708, 48706 and 48732 (located farther away from major cities). However, in addition to ZIP codes identified by Dai and Oyana [31], this study also pinpointed elevated levels of breast cancer in ZIP codes 48880 and 48618, located away from environmental exposure (point source pollution and floodplains of major rivers) and major communities. Elevated rates of breast cancer in ZIP codes 48618, 48880, and 48734 located farther away from the rivers and point source pollution may partly be due to the edge effect or missing data. High odds ratio/incidence rates of breast cancer in ZIP code 48880, located next to the reference ZIP code 48883, could potentially be due to the differences in demographic characteristics in these areas. Specifically, the population residing in ZIP code 48880 is more diverse (e.g., 76.0% White, 15.6% Black, 5.2% Hispanic, 1.0% Native American, 0.4% Asian, $35,176 median household income) with substantially higher presence of Blacks and Hispanics and significantly lower median household income than those residing in the ZIP code 48883 (e.g., 95.9% White, 0.4% Black, 1.8% Hispanic, 0.7% Native American, 0.2% Asian, $42,434 median household income). Additionally, the Michigan Public Health Institute cancer statistics report shows that the Midland and Saginaw counties (which contain ZIP codes 48640, 48603, 48734, 48880, 48618, and 48732) had higher age-adjusted incidence rates of breast cancer during the time period from 1994 to 2003 compared to incidence rates in the Bay county (located farther away from the Tittabawassee River) [30].

An increased risk of lung cancer was found in population living in ZIP codes 48601, 48706, 48708, 48732, 48602, and 48603. These ZIP codes are located within the area of concentrated industrial activity, responsible for releases of chemicals that have a potential to cause a lung cancer. These findings are consistent with results from previous studies of lung cancer and environmental pollution, suggesting that populations living in close proximity to industry experience greater risk of having lung cancer [20]. In addition, earlier report on cancer burden in central Michigan also pinpointed higher age-adjusted incidence rates of lung cancer in Bay county (which contains ZIP codes 48706, 48708, and 48732) compared to Midland and Saginaw counties [30]. 

Statistical and spatial analysis methods detected clusters of breast and lung cancer and confirmed study hypothesis of increased cancer risk associated with environmental exposure. Particularly, the odds ratio analysis found statistically significant association between risk of breast and lung cancer and exposure to environmental pollution. Results of exposure analysis suggest that female residents living in ZIP codes located in close proximity to the river have 1.5 to 2 times higher risk of having breast cancer than those who live farther away; the residents living in ZIP code nearby the point source pollution and major roads also have 1.5 to 2 times higher risk of having lung cancer than those who live farther away. 

Disease clustering provided further evidence of presence of clusters of breast and lung cancer in population residing in ZIP codes located in close proximity to environmental contamination. Specifically, Local Moran's test detected clusters of lung cancer among residents living in ZIP codes 48732 and 48708. These findings are in agreement with results of the Turnbull's method and Lawson and Waller score test that found similar spatial clusters of increased lung cancer incidence rates. Besides, two spatial clusters of breast cancer were detected in ZIP codes 48640 and 48603 using Turnbull's method. All of these detected clusters are located in close proximity to sources of environmental contamination. 

The study identified presence of environmental contaminants in the study area that have a potential to cause breast and lung cancer within at-risk population. These include arsenic, benzene, chromium, dichloromethane, naphthalene, nickel, TCE, and PAHs.

Despite the expectations, there were no clusters of breast cancer found around locations of industrial facilities selected as potential sources of increased risk of breast cancer. Interestingly, recent studies conducted in central Michigan suggest that there are dioxin-like toxins in Saginaw River watershed (including the Tittabawassee and Shiawassee Rivers, see Figure 1) [41, 42]. These studies found that concentrations of total PCBs (identified as animal mammary carcinogens and estrogen mimics with reported evidence of an association with breast cancer) in surface sediments and floodplain soils of the Saginaw River watershed were higher in samples collected from Middle Ground Island in the Saginaw River (situated in close proximity to the former landfill site collecting PCB-containing wastes) and from subsurface sediments in the mouth of Saginaw Bay (found in close proximity to the wastewater treatment plants and other industrial sources of environmental pollution). Although our study has detected spatial clusters of breast cancer in ZIP codes 48706 (located in the Middle Ground Island in the Saginaw River) and 48732 (located at the mouth of the Saginaw River which is in close proximity to point source pollution) further evidence is required to understand these clusters.

For lung cancer, this study detected clusters of lung cancer in close proximity to industrial facilities. The majority of these facilities were reported to be operationally active in 2002, which implies possible continuing impacts of releases from these facilities on the study population. The major industry types of these facilities include petroleum, wood, and cement production, which is in agreement with previous studies that demonstrated an increased risk of lung cancer in close proximity to petroleum and chemical industry [20, 43]. Reported carcinogenic compounds released from these facilities include arsenic, benzene, chromium, dichloromethane, naphthalene, nickel, TCE, and PAHs, identified in recent literature as known or suspected carcinogens that may cause a lung cancer [16, 17, 19, 21–26].

An assessment of the relative importance of socioeconomic factors suggests the significant importance of two risk factors: race and residency. Specifically, populations of Hispanic race/ethnicity (for both breast and lung cancer) and residency at the same location (for breast cancer) are more likely to be susceptible to the risk of cancer. These findings are consistent with results of previous studies suggesting differences in incidence of breast cancer based on ethnicity, and years of residence at the same location, and differences in the risk for lung cancer between ethnic groups [1, 9, 11–13, 22, 24].

## 4. Conclusions

Findings from this study support the hypothesis that there is a spatial association between the risk of cancer and environmental exposure. The study period of 14 years, although it could be longer than 20 years in some cases, generally captures the latency of cancer, further provided evidence on the effects of long-term exposure to air pollution on lung cancer. Geographic locations near major rivers were associated with high risk of breast cancer, while spatial clusters of lung cancer were found near point source pollution and major highways. The study identified the presence of environmental contaminants with a potential to explain increased risks of breast and lung cancer. Although this study did not provide specific evidence of contribution of identified industrial facilities to elevated levels of breast cancer, it confirmed the role of point source pollution in explaining the spatial variability of lung cancer. 

Previously found clusters of breast cancer in the study area were determined based on proximity to soil dioxin contamination, without taking into account risk of cancer in relation to exposure from point source pollution [31]. The new set of environmental risk factors (e.g., industrial facilities, major rivers, and highways) provided new evidence of spatial association between environmental exposure and risk of cancer. Preliminary findings also show potential sources of pollution that were associated with increased cancer incidence. However, there is an urgent need to determine the connection of cancer disease and environmental exposure using high-quality exposure data obtained from field measurements. Future studies should consider the contribution of regional and nonpoint sources of environmental contamination to the risk of breast and lung cancer. Furthermore, a larger sample size of cancer data could substantially benefit results of statistical and spatial analysis methods, specifically could significantly improve results of discriminant analysis of risk areas of cancer. Also, further evaluation of individual risks and socioeconomic characteristics of the population living in areas with high cancer rates may offer additional insights. Specifically, lung cancer patients already exposed to environmental pollution could be further burdened with smoking habits in the population.

The results of this study are useful to researchers and governmental agencies involved in regulation, control, and monitoring of environmental contamination within the floodplains of the Tittabawassee and Saginaw Rivers. Specifically, governmental agencies may consider control and monitoring of pollutants reported in releases of industrial facilities found to be associated with increased risk of lung cancer. Monitoring results could serve as the basis for the development of regulatory sanctions of pinpointed pollutants. These findings are also valuable for manufacturers to consider when conducting reassessment of production processes, in order to avoid or minimize releases of identified carcinogenic chemicals into the environment.

## Figures and Tables

**Table 1:** Industrial facilities identified as sources of carcinogenic releases that have a potential to cause a breast cancer.

ZIP code	Company name	X	Y	Industry type	Release type	Reported carcinogenic chemical(s)
48601	Delphi Saginaw Steering Systems	6039325.7	10957.3	Motor Vehicle Parts and Accessories	Multiple	Dichloromethane,
						trichloroethylene
48601	Safety-Kleen Systems	6038970.8	16378.6	Business Services	Multiple	Polycyclic aromatic
						hydrocarbons
48640	DOW Company Midland	6012861.0	31246.7	Multiple	Multiple	1,2-Butylene oxide, 1,1,2,2-
						Tetrachloroethane, 1,2-
						Dichloroethane, 1,2-
						Dichloropropane, benzene,
						carbon tetrachloride,
						chloroform, chloroprene,
						dichloromethane, dioxins,
						ethylene oxide,
						trichloroethylene
48642	Quantum Composites Inc.	6015892.6	32664.5	Custom Compound Purchased Resins	Multiple	Dichloromethane
48655	Thompson Marine Products Inc.	6018281.7	−531.6	Boat Building and Repairing	Toxics	Dichloromethane,
						trichloroethylene
48706	Marathon Petroleum Co LLC Bay City MI Terminal	6040477.5	33695.15	Petroleum Bulk Stations and Terminals	Multiple	Polycyclic aromatic
						hydrocarbons, benzene

**Table 2:** Industrial facilities identified as sources of carcinogenic releases that have a potential to cause a lung cancer.

ZIP code	Company name	X	Y	Industry type	Release type	Reported carcinogenic chemical(s)
48601	Delphi Saginaw Steering Systems	6039325.7	10957.3	Motor Vehicle Parts and Accessories	Multiple	Chromium,
						dichloromethane,
						nickel, trichloroethylene
48601	Means Industries Inc Saginaw Facility	6034205.5	10522.6	Automotive Stampings	Multiple	Chromium, nickel
48601	Safety-Kleen Systems	6038970.8	16378.6	Business Services	Multiple	Polycyclic aromatic
						hydrocarbons
48640	DOW Company Midland	6012861.0	31246.7	Multiple	Multiple	1,2-Dichloroethane,
						benzene, chromium,
						dichloromethane,
						dioxins, naphthalene,
						trichloroethylene
48642	Quantum Composites Inc.	6015892.6	32664.5	Custom Compound Purchased Resins	Multiple	Dichloromethane
48655	Thompson Marine Products Inc.	6018281.7	−531.6	Boat Building and Repairing	Toxics	Dichloromethane,
						trichloroethylene
48706	Marathon Petroleum Co LLC Bay City MI Terminal	6040477.5	33695.15	Petroleum Bulk Stations and Terminals	Multiple	Benzene,
						naphthalene,
						polycyclic aromatic
						hydrocarbons
48706	Straits Wood Treating	6040079.1	34216.1	Wood Preserving	Toxics	Arsenic, chromium
48708	General Motors Corp Power Train Bay City	6040442.6	32428.1	Motor Vehicle Parts and Accessories	Multiple	Chromium, nickel
48732	Essroc Cement Corp.	6042422.4	33446.6	Cement, hydraulic	Multiple	Chromium

**Table 3:** The odds ratio of having a breast/lung cancer and distribution of cancer incidence rates by ZIP codes, 1989–2002.

ZIP code	Cancer cases		Population		Incidence rate		Confidence interval		Odds Ratio		Confidence interval	
	(age 15 years and over)		(age 15 years and over)		per 10 K		for % rate				for Odds Ratio	
	Breast	Lung	Female	Total	Breast	Lung	Breast	Lung	Female	Total	Breast	Lung

48415	92	86	3827	7467	240	115	2.4 ± 0.48	1.15 ± 0.24	1.33	1.14	0.944–1.876	0.813–1.593
48457	69	77	3266	6532	211	118	2.11 ± 0.49	1.18 ± 0.26	1.17	1.16	0.811–1.677	0.825–1.644
48601	436	632	19205	34336	227	184	2.27 ± 0.21	1.84 ± 0.14	1.25	1.83^1^	0.939–1.677	1.394–2.405
48602	324	388	13344	25231	243	154	2.43 ± 0.26	1.54 ± 0.15	1.34	1.53^1^	1.000–1.807	1.153–2.107
48603	516	440	17399	32175	297	137	2.97 ± 0.25	1.37 ± 0.13	1.65^1^	1.35^1^	1.238–2.202	1.026–1.787
48604	128	106	4996	9491	256	112	2.56 ± 0.44	1.12 ± 0.21	1.42^1^	1.1	1.026–1.967	0.798–1.524
48611	52	50	2375	4634	219	108	2.19 ± 0.59	1.08 ± 0.3	1.21	1.07	0.820–1.783	0.727–1.560
48616	62	68	3072	6028	202	113	2.02 ± 0.5	1.13 ± 0.27	1.11	1.11	0.767–1.614	0.782–1.578
48618	58	51	2074	4087	280	125	2.8 ± 0.71	1.25 ± 0.34	1.55^1^	1.23	1.064–2.270	0.844–1.804
48623	87	60	4409	9883	197	61	1.97 ± 0.41	0.61 ± 0.15	1.09	0.60^2^	0.769–1.538	0.414–0.858
48626	45	53	2324	4528	194	117	1.94 ± 0.56	1.17 ± 0.31	1.07	1.16	0.713–1.596	0.794–1.685
48640	421	334	13339	25955	316	129	3.16 ± 0.3	1.29 ± 0.14	1.76^1^	1.27	1.316–2.355	0.960–1.688
48642	178	146	12610	23949	141	61	1.41 ± 0.21	0.61 ± 0.1	0.77	0.60^2^	0.566–1.056	0.440–0.815
48650	71	83	3062	6093	232	136	2.32 ± 0.53	1.36 ± 0.29	1.28	1.35	0.893–1.841	0.961–1.893
48655	62	72	2651	5195	234	139	2.34 ± 0.58	1.39 ± 0.32	1.29	1.37	0.891–1.877	0.968–1.947
48657	84	92	3222	6463	261	142	2.61 ± 0.55	1.42 ± 0.29	1.45^1^	1.41^1^	1.019–2.051	1.011–1.966
48706	407	573	17269	32814	236	175	2.36 ± 0.23	1.75 ± 0.14	1.3	1.74^1^	0.974–1.745	1.320–2.282
48708	285	372	11973	22896	238	162	2.38 ± 0.27	1.62 ± 0.16	1.32	1.61^1^	0.977–1.775	1.218–2.135
48732	143	142	5201	9640	275	147	2.75 ± 0.44	1.47 ± 0.24	1.53^1^	1.46^1^	1.108–2.105	1.072–1.989
48734	110	59	3265	5995	337	98	3.37 ± 0.62	0.98 ± 0.25	1.88^1^	0.97	1.349–2.630	0.673–1.399
48880	86	69	2594	8090	332	85	3.32 ± 0.69	0.85 ± 0.2	1.85^1^	0.84	1.307–2.625	0.590–1.195
48883	52	57	2861	5623	182	101	1.82 ± 0.49	1.01 ± 0.26	1	1	reference	reference

^1^significant positive association between exposure and outcome; ^2^significant negative association between exposure and outcome.

**Table 4:** Distribution of lung cancer clusters by location of industrial facilities according to Lawson and Waller score test.

Industrial facility	ZIP code	Test statistic	*P*-value (9 999 simulation runs)	Nominal *P*-value
Delphi Saginaw Steering Systems	48601	9.47265	.000100*	.000000
Means Industries Inc Saginaw Facility	48601	8.25636	.000100*	.000000
Safety-Kleen Systems	48601	10.92670	.000100*	.000000
DOW Company Midland	48640	−9.52227	1.000000	1.000000
Quantum Composites Inc.	48642	−9.11357	1.000000	1.000000
Thompson Marine Products Inc.	48655	1.441800	.169400	.074679
Marathon Petroleum Co LLC Bay City MI Terminal	48706	7.625880	.000100*	.000000
Straits Wood Treating	48706	7.746250	.000100*	.000000
General Motors Corp Power Train Bay City	48708	7.564160	.000100*	.000000
Essroc Cement Corp.	48732	6.538380	.000100*	.000000

*α* = 0.01; number of Monte Carlo simulations = 9 999, *statistically significant
